# Male-dependent resistance to *Spiroplasma*-induced cytoplasmic incompatibility

**DOI:** 10.1098/rsos.250545

**Published:** 2025-06-18

**Authors:** Marie Pollmann, Ronja Reinisch, Lea von Berg, Molly Avidan King, Marina Geiselmann, Lena-Maria Käppeler, Raz Leibson, Natascha Traub, Johannes L. M. Steidle, Yuval Gottlieb

**Affiliations:** ^1^ Koret School of Veterinary Medicine, The Robert H. Smith Faculty of Agriculture, Food and Environment, Hebrew University of Jerusalem, Rehovot, Israel; ^2^ Department of Chemical Ecology, Institute of Biology, and KomBioTa – Centre of Biodiversity and Integrative Taxonomy, University of Hohenheim, Stuttgart, Germany; ^3^ Department of Insect Symbiosis, Max-Planck-Institute for Chemical Ecology, Jena, Germany; ^4^ Evolutionary Biology of Invertebrates, Institute fo Evolution and Ecology, University of Tübingen, Tübingen, Germany

**Keywords:** cytoplasmic incompatibility, host resistance, *Spiroplasma* phenotypes, *Lariophagus distinguendus* clades, bacterial cross-transfer

## Abstract

Cytoplasmic incompatibility (CI) caused by bacterial endosymbionts is an embryonic developmental failure between infected host males and uninfected females. Although even closely related hosts can have different CI phenotypes, little is known on the resistance mechanism in non-susceptible hosts. The parasitoid wasp species complex of *Lariophagus distinguendus* encompasses at least three species, termed clades A, B and C. All three species contain strains infected with the endosymbiotic bacterium *Spiroplasma*, which causes CI in clade A. We studied the relatedness of *Spiroplasma* in the species complex, the occurrence of CI in selected strains, and the effect of host strain and sex on CI induction. According to multi-locus sequence typing, all host species carry the same *sDis* strain. CI was absent in strains of clades B and C. Cross-transferring *sDis* revealed a male-dependent CI resistance in clade B. Together, this suggests a single infection event in the ancestor of all *L. distinguendus* clades. Some *L. distinguendus* strains are susceptible to CI, others are resistant. At least in one strain, resistance to CI is male-dependent, as theory predicts, supporting male-dependent traits as drivers for loss of CI-inducing bacteria. These results facilitate future studies on the mechanism of *Spiroplasma*-induced CI and its resistance.

## 1. Introduction

Cytoplasmic incompatibility (CI), the most common bacterial-induced reproductive manipulation in arthropods [1], is defined as incompatibility between sperm of infected males and eggs of females that are either uninfected or carry a bacterial strain that is incompatible to the strain infecting the males [1,2]. As a result, diploid offspring fail to develop, or only haploid individuals emerge in organisms with haplodiploid sex determination such as Hymenoptera [1–3]. Interestingly, for the same endosymbiont, the form of reproductive manipulation and its expression level can vary depending on host genetic background (e.g. [4–11]). Moreover, the ability of one endosymbiont to induce different phenotypes even in the same host species has been documented [12]. Many cases of host-dependent modulation of CI strength have been linked to variability in the access of the endosymbiont to developing sperm, indicated by its presence or absence, its specific localization, and its titre in the testes during sperm development [13–16]. Accordingly, theoretical models expect the resistance to CI to be favoured in males rather than females [17–20]. However, there is little experimental evidence to support this hypothesis.

CI is induced by *Wolbachia* (Alphaproteobacteria), the most widespread CI inducer [1,2], *Candidatus* Cardinium hertigii (Bacteroidota) [21,22], *Candidatus* Mesenet longicola (Alphaproteobacteria) [23,24], *Rickettsiella* (Gammaproteobacteria) [25] and *Rickettsia* (Alphaproteobacteria) [26]. Recently, *Spiroplasma* (Mollicutes) of the *ixodetis* clade was demonstrated to induce CI in *Lariophagus distinguendus* (Förster, 1841) (Hymenoptera : Chalcidoidea : Pteromalidae) [27]. Members of the *S. ixodetis* clade readily colonize novel hosts, even if these are phylogenetically distant from their original hosts [28], leading to incongruent endosymbiont and host phylogenies [29]. Some members of the *ixodetis* clade have no discernible effect on their hosts, but many act as pathogens or reproductive manipulators [30–33].

The pteromalid wasp *L. distinguendus* is a parasitoid of coleopteran larvae [34]. In recent years, *L. distinguendus* was revealed as a complex of at least three species, provisionally referred to as clades A, B and C, which are separated by various isolating barriers, including different host preferences [35–38]. Endosymbiont-induced CI acts as unidirectional isolation barrier between males of clade A strains (formerly DB-lineage) and females of a strain belonging to clade C (formerly GW-lineage) [36], as well as females from another strain of clade A [38]. *Spiroplasma*, termed *sDis*, infecting the clade A strains dbSTU-D1 (STU) and dbRAV-D1 (RAV) was identified as the cause for this CI [27,38]. Other strains from clades A, B and C of *L. distinguendus* also carry *Spiroplasma* [38] (also see table 1) but it is unknown if these infections are associated with CI in clades B and C. Nevertheless, no CI occurred in crosses between STU (clade A) and the clade B strain dbCAN-D1 (CAN). Given previous data on *Spiroplasma* prevalence and CI induction in *L. distinguendus* and the theoretical prediction mentioned above, we hypothesized that CI phenotype may vary among the wasp clades and aimed to determine whether the expected variation is symbiont- or host-dependent, and to identify specific factors that may contribute to our findings. As a first step, we also aimed to examine the relatedness of the *Spiroplasma* strains in *L. distinguendus*.

**Table 1.** *L. distinguendus* strains used in this study with host species (db: drugstore beetle, gw: granary weevil), collection site, year of collection, and infection status.

clade^a^	strain	abbreviation	host	collection site	year of collection	infection^b^
A	dbBIR-D1	BIR	db	Stuttgart-Birkach, Germany	2011	uninfected by nature
A	dbRAV-D1	RAV	db	Ravensburg, Germany	2008	*Spiroplasma*
A	dbSTU-D1	STU	db	Stuttgart, Germany	2007	*Spiroplasma*
B	dbCAN-D1	CAN	db	Stuttgart-Bad Cannstatt, Germany	2017	*Spiroplasma*
B	dbWAG-N1	WAG	db	Wageningen, The Netherlands	2011	*Spiroplasma*
C	gwPFO-D1	PFO	gw	Pforzheim, Germany	2005	*Spiroplasma*, *Wolbachia*^c^
C	gwSWD-D1	SWD	gw	Schwieberdingen, Germany	2018	*Spiroplasma*, *Wolbachia*

^a^ Designations taken from [38].

^b^ As stated in appendix of [38].

^c^ This infection has no noticeable effect on its host, as determined by [36,38].

For this, we tracked the phylogeny and phenotype of other *Spiroplasma* in the *L. distinguendus* species complex using multi-locus sequence typing (MLST) and tested CI induction in strains representing clades B and C, as well as the influence of host strain and host sex on CI presence.

We found that *sDis* strains are identical and thus likely infected the *L. distinguendus* species complex prior to speciation events. Additionally, we demonstrated that some host species within the species complex are susceptible, while others are resistant to *Spiroplasma*-induced CI and that this resistance is a male-dependent trait, supporting theoretical predictions that the evolution of CI resistance is favoured in males.

## 2. Material and methods

### 2.1. Insects

Some of the wasp strains used in this study were collected with baits consisting of plastic tubes containing koi pellets infested with larvae of *Stegobium paniceum* (L., 1758), which were distributed to volunteers and placed in locations accessible to the wasps present in the environment. Other strains were acquired by chance in the proximity of stored products infested by one of their host species in grain stores or household pantries. Adult wasps hatched from the baits or collected as adults were placed on the appropriate host species to establish the cultures which have been maintained since their date of collection (see table 1 for collection site and year). Wasp strains with granary weevils as hosts were reared on 4-week-old larvae of *Sitophilus granarius* (L., 1758) infesting wheat grains, whereas wasps with drugstore beetles as hosts were reared on 6-week-old larvae of *S. paniceum* in wheat grains or koi pellets (Hikari Friend, Kamihata Fish Industry Group, Kyorin Corporation, Japan). All wasp strains were reared at 26°C and 45% RH, with a natural light : dark cycle in Petri dishes or honey jars with a ventilated lid (diameter 12 cm, height 16 cm). Wasps intended for experiments were isolated from the infested substrate in the pupal stage (see table 1 for wasp strains used in this study and their preferred host species). To maintain drugstore beetle cultures, 80 g of koi pellets in honey jars were inoculated with approximately 1 g adult beetles and kept at 27°C and 45% RH. For granary weevil cultures, 2.7 g adult weevils were placed in honey jars containing 200 ml of wheat grains (*Triticum aestivum* L.) moistened with 1 ml water per 40 g wheat grains beforehand. The honey jars with the infested wheat grains were initially kept at 25°C for 3 weeks, at 20°C for the subsequent week, and at 15°C with approximately 45% RH during the stage at which they were suitable for parasitization. Adult weevils were removed after the first week. Beetles and weevils not used as hosts for wasp cultures served to establish the next generation. For both host species, the light : dark cycle was either natural or 16 h L : 8 h D.

### 2.2. Multi-locus sequence typing

The diversity of *Spiroplasma* infecting the *L. distinguendus* species complex was investigated by MLST of *Spiroplasma* from the wasp strains STU, RAV (clade A), WAG, CAN (clade B) and PFO (clade C). DNA from 10 male and 10 female wasps per strain was extracted by crushing single wasps in 10 µl of a lysis buffer containing 10 mM Tris, 0.5 mM EDTA pH 9.0, 3 mg Proteinase K and 5 µl Igepal (Sigma-Aldrich Products Ltd, Rehovot, Israel) and collecting each sample with an additional 30 µl of lysis buffer, followed by incubation at 65°C for 15 min and 95°C for 10 min and finally centrifugation at 10 000 × *g* for 1 min to collect the supernatants subsequently used as samples. Samples were subjected to nested PCR with primers for 16S rRNA, *dnaK*, *EpsG*, *gyrA* and *rpoB* following [29]. The reaction mix for the first amplification consisted of 12.5 µl Promega GoTaq^®^ Green Master Mix 2X (Promega, Madison WI, USA), 1 µl per primer and 8.5 µl double distilled water for 2 µl template. 1 µl PCR product from the first PCR served as template for the second amplification, using the same amount per reagent with the volume of double distilled water increased to 9.5 µl. PCR products of four samples per strain were cleaned using ExoSAP-IT™ (Thermo Fisher Scientific, Waltham MA, USA) and bidirectionally sequenced at Macrogen Europe (Amsterdam, The Netherlands). Consensus sequences per *Spiroplasma* strain and gene were created with MEGA version 11 [39] and BioEdit version 7.2.5 [40] by manual analysis and editing. Heterozygous positions were assigned ambiguous characters according to the IUPAC nucleotide ambiguity code [41]. Amplification success was confirmed by submitting all sequences to BLAST [42]. Alignments were created for each gene with the sequences of *Spiroplasma* from *L. distinguendus* and of three *S. ixodetis* strains retrieved from GenBank [43] (see electronic supplementary material, table S1, for accession numbers) in MAFFT v7.511 [44] with the L-INS-I algorithm [45] and curated using Gblocks implemented in Phylogeny.fr with the option ‘Allow gap positions within the final blocks’ [46,47]. Using Virtual Ribosome v. 2.0 [48], the sequences were transcribed into amino acid sequences with translation table 4 (*Spiroplasma*) to determine reading frames and exclude the presence of unexpected stop codons and gaps. A concatenated matrix of all sequences per isolate was created in MEGA and the genes were analysed jointly after confirming partition homogeneity (*p* = 1.0) in PAUP* version 4.0a (build 169) [49]. The most suitable partitioning schemes and nucleotide substitution models were determined with ModelFinder [50] (see electronic supplementary material, table S2, for results) and implemented by IQ-TREE version 1.6.12 to compute a maximum likelihood phylogenetic tree using ultrafast bootstrapping [51,52] and utilizing the edge-proportional partition model (-spp [53]) and the Bayesian information criterion [54] alongside standard parameters. Visualization and final formatting of the phylogenetic tree were conducted in MEGA.

### 2.3. Testing for *sDis* infection

For DNA extraction, a nexttec 1-step tissue and cells kit (nexttec Biotechnologie GmbH, Hilgertshausen, Germany) and a DNeasy Blood and Tissue kit (Qiagen, Hilden, Germany) were used following the manufacturers’ instructions.

*sDis* infection was tested by PCR on *dnaA* using the primers ApDnaAF1 (5′-ATT CTT CAG TAA AAA TGC TTG GA-3′ [31]) and ApDnaAR1 (5′-ACA CAT TTA CTT CAT GCT ATT GA-3′ [31]). PCR conditions were 95°C for 4 min and 35 cycles of 95°C for 30 s, 55°C for 30 s, and 72°C for 1 min or 95°C for 4 min, 35 cycles of 95°C for 30 s, 52°C for 30 s and 72°C for 1 min and a final elongation of 72°C for 5 min. The reaction mix consisted of 12.5 µl ROTI^®^Pol TaqS Red-Mix (2×) (Carl Roth GmbH+Co. KG, Karlsruhe, Germany), 1 µl per primer and 9.5 µl double distilled water per 1 µl template.

### 2.4. Antibiotic treatment generating endosymbiont-free lines

Tetracycline-treated (tetracycline hydrochloride, Sigma-Aldrich Chemie GmbH, Taufkirchen, Germany), endosymbiont-free lines of the strains STU (clade A), CAN (clade B) and SWD (clade C) (STU_tet_, CAN_tet_, and SWD_tet_) were established and maintained by placing wasps in Petri dishes containing filter paper and a piece of cotton wool soaked in a tetracycline solution (1 mg tetracycline and 100 mg sucrose per 1 ml water) for 24 h. Subsequently, they were transferred to wheat grains or koi pellets infested with their respective hosts. After at least three generations, the absence of endosymbionts was confirmed by PCR (see above) in randomly selected individuals. To minimize the risk of using individuals falsely tested as negative, experiments were only started after all tested individuals from at least one generation of tetracycline-treated lines had been tested negative.

### 2.5. *sDis* transfer between strains

*sDis*-containing haemolymph was transferred from STU (clade A) females into CAN_tet_ (clade B) females and vice versa and naturally endosymbiont-free BIR (clade A) females [38] were injected with STU-*sDis*-containing haemolymph. Using a procedure established by a previous study [27], haemolymph was collected from the donor females with a glass capillary and directly injected into the recipient females using an Eppendorf FemtoJet 5247 (Eppendorf AG, Hamburg, Germany). Injected females were transferred to batches of 5 g of koi pellets containing *S. paniceum* larvae. The first host batch was discarded and replaced after 5 days because injected females do not transmit *sDis* to their offspring during the first few days [27]. Females were examined for *sDis* after their deaths (see above) and their offspring were tested for CI (see below).

### 2.6. Testing for cytoplasmic incompatibility

CI was investigated in crossing experiments. Single males and females were placed together and observed until they had mated or for a maximum of 20 min. Afterwards, mated females were transferred to host substrates consisting in 5 g of koi pellets with drugstore beetle larvae for CAN (clade B), STU (clade A), and BIR (clade A), and 10 g of granary weevil-infested wheat grains for SWD (clade C), where they remained until their deaths. The resulting offspring were counted and sexed 4 or 5 weeks later. CI was assumed when female offspring in crosses between *sDis*-carrying males and endosymbiont-free females were significantly reduced because in haplodiploids like *L. distinguendus*, only the diploid females require fertilization and are therefore decreased as a result of CI disrupting successful fertilization, whereas the haploid males remain unaffected (e.g. [3,55]).

The occurrence of CI was investigated in CAN and SWD by performing intrastrain crosses with males and females of tetracycline-treated, uninfected lines and untreated, infected lines in all possible combinations. To test for CI in transfection experiments, F1 male offspring of injected females which were positive for *sDis* were mated to endosymbiont-free females of the recipient strain. The corresponding controls were tetracycline-treated males and females of the same strains in the cross-transfers of *sDis* between CAN and STU and negative F1 male offspring of injected BIR females for the transfer of *sDis* to BIR. Only the couples for which copulation was recorded were used for the remaining experiment to ensure any observed effects were not due to the lack of mating. After mating, F1 males were tested for *sDis* and the resulting F2 offspring numbers were analysed.

### 2.7. Testing for sex-specific cytoplasmic incompatibility resistance in CAN

To investigate sex-specific CI resistance, a STU-*sDis*-infected CAN line (clade B) was established. Haemolymph was transferred by injection (see above) from STU females (clade A) to CAN females. Injected CAN females were mated to CAN males and tested for *sDis* after their deaths (see above). F1 female offspring of infected mothers were then mated to CAN males, allowed to oviposit and tested for infection after they had died. Using the descendants of *sDis-*positive females, this procedure was continued over several generations, testing randomly selected individuals in every generation for infection until a STU-*sDis*-infected CAN line had been established. The ability of CAN females to resist CI was tested by comparing offspring numbers between crosses of uninfected CAN females and infected and uninfected STU males. The ability of males to resist CI induction was tested by comparing offspring numbers of crosses between uninfected and STU-*sDis*-infected CAN males with uninfected STU_tet_ females.

### 2.8. Statistical analyses

Statistical analyses were conducted using R v. 4.4.0 implemented in RStudio v. 2024.04.0.735 [56,57], with the additional packages multcomp [58], lme4 [59] and lmerTest [60]. Data of F1 female offspring from intrastrain crosses of tetracycline-treated and infected individuals of CAN and SWD as well as total F1 offspring numbers of SWD were normally distributed and therefore analysed using linear models. Comparisons for non-normally distributed F1 male offspring numbers of these crosses and total offspring numbers of CAN were conducted using generalized linear models with the family most suitable for the data. These models were followed by Tukey tests for single comparisons. F2 offspring numbers in crossings between F1 male offspring of injected *sDis*-positive females or control males and CAN_tet_ and STU_tet_ females were analysed using generalized linear mixed models (family = negative binomial), since data were not normally distributed. Host age, which can have an effect on host suitability due to older hosts being larger (e.g. [61,62]), was included as random factor in the analysis of the *sDis* transfer experiments between STU and CAN to correct for distorting effects. All F1 offspring numbers in crosses between STU_tet_ females and infected or uninfected CAN males as well as total offspring numbers in crosses of uninfected CAN females and infected and uninfected STU males adhered to normal distribution and were compared with Welch two sample *t*-tests followed by Bonferroni corrections, whereas F1 female and male offspring numbers in the latter crosses were compared using Wilcoxon rank sum tests with continuity correction followed by Bonferroni correction because these data were non-normally distributed. The same was true for all F2 offspring numbers in the crosses between BIR females and F1 male offspring of BIR females injected with STU-*sDis*. Graphs and figures were constructed with R in RStudio, implementing the packages ggplot2 [63], extrafont [64] and patchwork [65], as well as with Adobe Photoshop version 26.0.0 (Adobe Inc.).

## 3. Results

### 3.1. *Spiroplasma* in the *Lariophagus distinguendus* complex are identical

**Figure 1.**
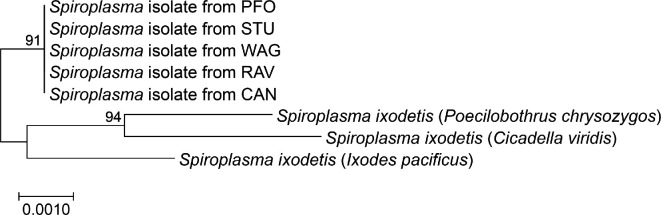
Maximum likelihood phylogenetic tree generated from MLST of *sDis* from *L. distinguendus* strains RAV and STU (clade A), CAN and WAG (clade B), and PFO (clade C) and known *S. ixodetis* isolates from NCBI, with their host species in parentheses. See electronic supplementary material, table S1, for accession numbers of *S. ixodetis* isolates taken from NCBI.

The identity of *Spiroplasma* in the *L. distinguendus* complex was studied using MLST in the strains STU, RAV (clade A), WAG, CAN (clade B) and PFO (clade C). All isolates are closely related to published *S. ixodetis* strains and belong to the same strain, *sDis*, with no detectable differences among them at the MLST gene level (figure 1; see electronic supplementary material, table S3, for NCBI accession numbers for sequences generated in this study).

### 3.2. Induction of cytoplasmic incompatibility by *sDis* in the *L. distinguendus* complex

**Figure 2.**
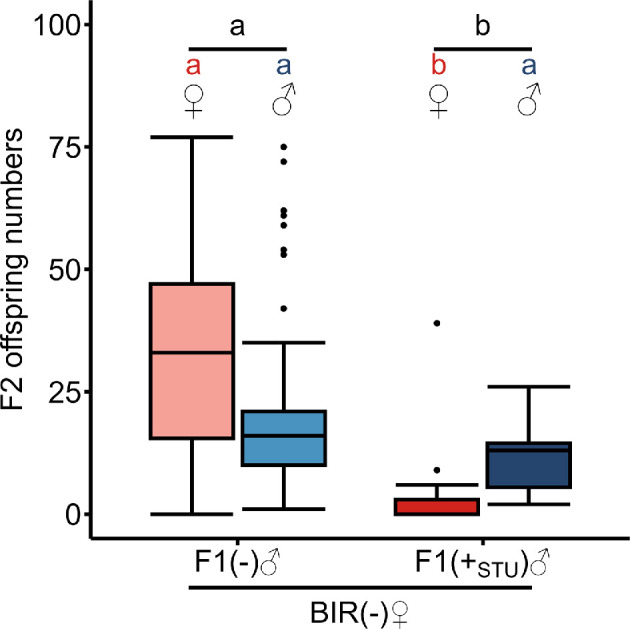
CI induction in the originally uninfected BIR (clade A). Numbers of female (red) and male (blue) F2 offspring of uninfected (−) (*n* = 163) and STU-*sDis*-infected (+_STU_) (*n* = 11) F1 BIR males. Lighter coloured boxes: crosses with uninfected F1 males, darker coloured boxes: crosses with infected F1 males. Different lower-case letters (red: female, blue: male, black: total offspring) indicate statistical differences at *p* < 0.05 (Wilcoxon rank sum tests followed by Bonferroni correction). Box and whisker plots: the boxes contain values within the interquartile range, medians are the middle horizontal lines, whiskers include values within a range across 1.5 times the interquartile range above the 75% and below the 25% quartiles, values outside of this range are shown as single points.

To study CI presence in representatives of the three clades, uninfected and *sDis*-carrying males and females of the *L. distinguendus* strains BIR (clade A), CAN (clade B) and SWD (clade C) were crossed in different combinations. BIR was uninfected when it was collected. Therefore, experiments were performed with BIR individuals artificially infected by injecting females with *sDis*-containing STU haemolymph. Approximately 30% (8 of 27) of the injected females later tested positive for *sDis* and 19% (11 of 57) of the male offspring of positive females inherited the *sDis* infection. F1 male offspring of these females were crossed to naturally uninfected BIR females. F2 female (*p* = 3.862 × 10^−5^ ***) and total offspring numbers (*p* = 6.365 × 10^−5^ ***) were significantly reduced in crosses with *sDis*-infected F1 males compared with uninfected F1 males (Wilcoxon rank sum tests with continuity correction, followed by Bonferroni correction). F2 male offspring numbers did not differ between crosses with positive and negative F1 males (*p* = 0.061 n.s., Wilcoxon rank sum test with continuity correction, followed by Bonferroni correction) (figure 2), suggesting induction of CI of the female mortality type, which encompasses the death of the diploid zygote [55,66].

**Figure 3.**
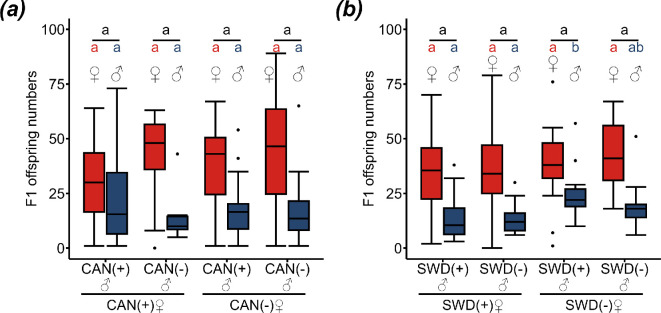
No CI induction in CAN (clade B) and SWD (clade C). Numbers of F1 female (red) and male (blue) offspring of infected (+) and uninfected (−) females and males of (*a*) CAN (CAN ♀ × CAN ♂: *n* = 20; CAN ♀ × CAN_tet_ ♂: *n* = 19; CAN_tet_ ♀ × CAN ♂: *n* = 20; CAN_tet_ ♀ × CAN_tet_ ♂: *n* = 20) and (*b*) SWD (SWD ♀ × SWD ♂: *n* = 22; SWD ♀ × SWD_tet_ ♂: *n* = 21; SWD_tet_ ♀ × SWD ♂: *n* = 21; SWD_tet_ ♀ × SWD_tet_ ♂: *n* = 21). Different lower-case letters (red: female, blue: male, black: total offspring) indicate statistical differences at *p* < 0.05 (LMs and GLMs followed by Tukey tests for multiple comparisons; see electronic supplementary material, tables S4–S9, for test statistics). Box and whisker plots: the boxes contain values within the interquartile range, medians are the middle horizontal lines, whiskers include values within a range across 1.5 times the interquartile range above the 75% and below the 25% quartiles, values outside of this range are shown as single points.

CI in CAN (clade B) and SWD (clade C) was tested by crossing males and females from naturally *sDis*-infected lines and tetracycline-treated, uninfected lines in all possible intrastrain combinations. There were no significant differences in female, male and total F1 offspring numbers (LMs and GLMs followed by Tukey tests; see electronic supplementary material, tables S4–S7 and S9, for test statistics) (figure 3) except for increased F1 male offspring numbers in the crosses between uninfected SWD females and males compared with the crosses with infected females (SWD_tet_ ♀ × SWD ♂ − SWD ♀ × SWD ♂: *p* = 0.001 **, SWD_tet_ ♀ × SWD ♂ − SWD ♀ × SWD_tet_ ♂: *p* < 0.001 ***, GLM, family = negative binomial, followed by Tukey tests; see electronic supplementary material, table S8 for test statistics) (figure 3*b*).

### 3.3. Resistance to *sDis*-induced cytoplasmic incompatibility is host-dependent

**Figure 4.**
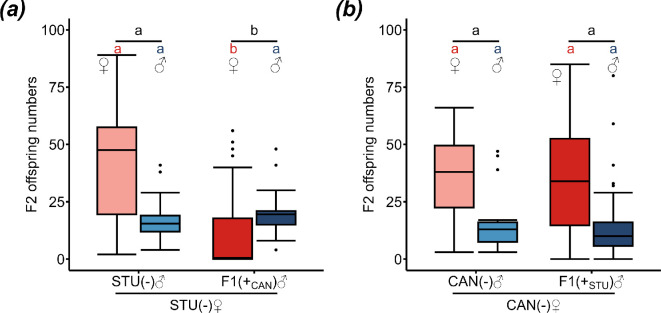
Host-dependent CI induction. Numbers of female (red) and male (blue) F2 offspring of uninfected females and males carrying *sDis* from another strain. (*a*) Crosses between uninfected (−) STU females and uninfected (−) control STU males (*n* = 26) or CAN-*sDis*-carrying (+_CAN_) F1 STU males (*n* = 28); (*b*) crosses between uninfected (−) CAN females and uninfected (−) control CAN males (*n* = 19) or STU-*sDis*-carrying (+_STU_) F1 CAN males (*n* = 108). Lighter coloured boxes: control crosses, darker coloured boxes: crosses with *sDis*-carrying F1 males. Replicate numbers per combination are given in brackets below the males. Different lower-case letters (red: female, blue: male, black: total offspring) indicate statistical differences at *p* < 0.05 (GLMMs, family = negative binomial, host age as random factor; see electronic supplementary material, tables S10–S15, for test statistics). Box and whisker plots: the boxes contain values within the interquartile range, medians are the middle horizontal lines, whiskers include values within a range across 1.5 times the interquartile range above the 75% and below the 25% quartiles, values outside of this range are shown as single points.

To reveal the factors affecting CI absence in *sDis*-infected strains, we tested if CI induction is host- or endosymbiont-dependent by cross-transferring *sDis* between STU (clade A), a strain showing CI and CAN (clade B), a strain without CI. In the transfer of *sDis* from CAN to STU, approximately 64% (23 of 36) of injected females tested positive for *sDis* and 21% (28 of 134) of the male offspring produced by the positive females were positive themselves. The reverse transfer yielded approximately 83% (29 of 35) positive injected females and 43% (108/251) of their male offspring also carried *sDis*. *sDis*-positive F1 males were mated to uninfected females of the same strain and the offspring numbers were compared with control crosses with *sDis*-negative males. F1 STU males infected with CAN-*sDis* sired significantly fewer F2 female (*p* = 0.001 **) and total (*p* = 3.38 × 10^−5^ ***) offspring in crosses with uninfected STU females than STU_tet_ males (GLMMs, family = negative binomial with host age as random factor; see electronic supplementary material, tables S10 and S12, for test statistics) (figure 4*a*). F2 male offspring did not differ between the crosses (GLMM, family = negative binomial with host age as random factor; see electronic supplementary material, table S11, for test statistics) (figure 4*a*). Therefore, CI of the female mortality type is induced in these crosses. By contrast, there were no differences between crosses of F1 STU-*sDis*-infected CAN males and CAN_tet_ males with CAN_tet_ females (GLMMs, family = negative binomial with host age as random factor; see electronic supplementary material, tables S13–S15, for test statistics) (figure 4*b*).

### 3.4. Resistance to *sDis*-induced cytoplasmic incompatibility is male-dependent

To test whether CI resistance in CAN (clade B) is a sex-dependent trait, uninfected CAN females were crossed with either infected or uninfected STU (clade A) males. In parallel, infected or uninfected CAN males were crossed with uninfected STU_tet_ females. Infected males were all carrying STU-*sDis*.

**Figure 5.**
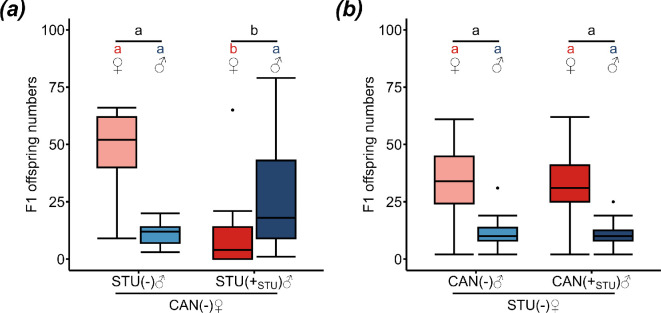
CI resistance is male-dependent. Numbers of female (red) and male (blue) F1 offspring of (*a*) uninfected (−) CAN females and uninfected (−) (*n* = 21) or STU-*sDis*-infected (+) (*n* = 21) STU males; (*b*) uninfected (−) STU females and uninfected (−) (*n* = 30) or STU-*sDis*-infected (+_STU_) (*n* = 15) CAN males. Lighter coloured boxes: crosses with uninfected males, darker coloured boxes: crosses with STU-*sDis-*infected males. Replicate numbers per combination are given in brackets below the males. Different lower-case letters (red: female, blue: male, black: total offspring) indicate statistical differences at *p* < 0.05 (Wilcoxon rank sum tests or Welch two sample *t*-tests followed by Bonferroni correction). Box and whisker plots: the boxes contain values within the interquartile range, medians are the middle horizontal lines, whiskers include values within a range across 1.5 times the interquartile range above the 75% and below the 25% quartiles, values outside of this range are shown as single points.

CAN females had significantly lower F1 female and total offspring numbers when crossed to STU-*sDis*-infected compared with uninfected STU males (female offspring: *p* = 1.071 × 10^−6^ ***, Wilcoxon rank sum test with continuity correction followed by Bonferroni correction; total offspring: *p* = 0.002 **, Welch two sample *t*‐test followed by Bonferroni correction), while the number of F1 males did not differ (*p* = 0.031 n.s., Wilcoxon rank sum test with continuity correction followed by Bonferroni correction) (figure 5*a*). By contrast, no differences were observed between the crosses of uninfected STU_tet_ females with either STU-*sDis* infected or uninfected CAN males (female offspring: *p* = 0.891 n.s., male offspring: *p* = 1 n.s.; total offspring: *p* = 0.890; Welch two sample *t*-tests followed by Bonferroni correction) (figure 5*b*).

## 4. Discussion

Here we show that *sDis* infecting the three clades of the *L. distinguendus* species complex most likely originated from a single infection event, which would have been followed by an infection loss in some strains of clades A and C. While all strains of clade A tested so far exhibited CI, at least one strain of clade B, and possibly clade C, evolved a mechanism to resist CI via a male-dependent trait.

### 4.1. *Spiroplasma* in the *L. distinguendus* complex originated from a single event infection event

MLST data revealed that all tested *L. distinguendus* strains across the three clades carried similar *sDis* strains. This strongly indicates that the common ancestor of *L. distinguendus* was infected by *sDis* in a single event. The alternative hypothesis, recent horizontal transmission of *sDis* between the infected strains, seems highly unlikely. Apart from the strains that were collected in the area of Stuttgart, Germany, the collection localities of several other strains were far away from each other in other parts of Germany or even in The Netherlands. So, many strains were not in direct contact with one another (see table 1 for collection sites of the examined strains).

This result is also in line with previously obtained crossing experiments between naturally *sDis*-infected CAN and STU individuals, for which no CI was observed. They demonstrate that *sDis* from these strains can prevent each other’s CI, i.e. that they are compatible with each other [27,38]. We also confirmed the result from a previous study that *sDis* belongs to the *Spiroplasma ixodetis* clade [27]. These findings agree with other host–*Spiroplasma* systems, where the endosymbionts in closely related hosts are themselves related (e.g. [67,68]). Generally, however, cospeciation or cocladogenesis between *Spiroplasma* and its hosts seems to be uncommon [29,33]. According to the current data, *sDis* was subsequently lost in most strains of clade C, several strains of clade A, but not in clade B.

### 4.2. *sDis* induces cytoplasmic incompatibility in clade A but not in clades B and C of the *L. distinguendus* complex

We performed crossing experiments to test for CI induction in representative strains of the *L. distinguendus* complex. First, we studied the induction of CI in clade A using the naturally uninfected BIR strain experimentally infected with *sDis* of the STU strain (both from clade A). Female and total offspring numbers were reduced in the CI test cross between STU-*sDis* infected F1 male offspring and uninfected females, Thus, BIR is susceptible to CI induced by STU-*sDis*. This also demonstrates the ability of *sDis* to infect organisms it did not have a recent relationship with and to induce CI like in its original hosts.

To study CI in clades B and C, we chose the naturally infected strains CAN and SWD, respectively, as representatives. In contrast to the experiments with BIR (clade A, see above), the numbers of female or total offspring were not reduced in any of the crossings. Obviously, the native *sDis* infections do not cause CI in either strain. This suggests either that the specific *sDis* strains of these hosts are unable to induce CI, as has been found for different *Wolbachia* strains (e.g. [69–73]), or that CAN and SWD have the ability to resist CI. Based on the results of our transfection experiments (see below) and as *sDis* seems to be identical in all *L. distinguendus* strains, we believe that the latter explanation is more likely. In fact, the evolution of resistance is considered a general theme in host–endosymbiont interactions involving reproductive manipulations, including CI [17,74,75]. This finding confirms that the CI phenotype indeed varies among the wasp clades, as hypothesized. Interestingly, SWD is also infected with *Wolbachia* like all strains of clade C (see table 1). Apparently, this *Wolbachia* infection is not causing CI either, or any other reproductive manipulation. The same result was previously shown for the strain PFO, which is also a member of clade C and also carries *Wolbachia* [36,38], also see table 1.

### 4.3. CAN (clade B) is resistant to cytoplasmic incompatibility induction by *sDis*

To study if CAN is resistant to CI, or if CAN-*sDis* is unable to induce CI, we performed transfection experiments between CAN and STU. *sDis* of STU (clade A) and CAN was transferred to tetracycline-treated, uninfected females of the opposite strains and infected F1 male offspring of these females were crossed to uninfected, tetracycline-treated females of their own strain. Whereas crosses between CAN-*sDis*-infected STU F1 males and uninfected STU_tet_ females had fewer F2 female and total offspring than controls, no effect was found for crosses with STU-*sDis*-infected CAN F1 males and CAN_tet_ females. Together with the finding that CI is absent in intrastrain crossings of CAN (see above), this demonstrates that CAN wasps are able to resist *sDis*-induced CI, regardless of the origin of *sDis*, showing that the variation of the CI phenotype in *L distinguendus* is in fact host-dependent. Host effects on endosymbiont phenotypes are known from various transfection and introgression experiments, with different reproductive manipulations in different hosts [6,8,9]. For endosymbiont-induced reproductive manipulations other than CI, incidences of host-dependent absence or active host resistance have been reported for a number of host species, predominantly for the male-killing phenotype (e.g. [10,18,76–80]). With respect to CI, its strength and occurrence have been shown to vary depending on host genetic backgrounds, often linked to endosymbiont presence or titre in specific host reproductive tissues [4,5,7,16,81].

A specific resistance factor against CI-inducing *Wolbachia* was found in *Nasonia vitripennis* (Walker, 1836), a member of the Pteromalidae like *L. distinguendus*. In this species, the titre of the *Wolbachia* strain *w*VitA is reduced by the maternal-effect nuclear gene *Wds* (*Wolbachia* density suppressor). Presumably, it blocks *Wolbachia* from entering the oocytes where usually a strong proliferation takes place [82]. No such correlation was detected between CI level and *sDis* titre in STU by a previous study [27]. However, this study only considered whole-body titres and did not address occurrence, localization and titres in host reproductive tissues, which are often associated with CI level [13–16,27,83]. Corresponding data for the resistant strain CAN are also unavailable. In *Drosophila*, artificial suppression of *Wolbachia*-induced CI was achieved by feeding substances associated with DNA repair and cell cycle timing to females, demonstrating a potential maternal effect on CI [84]. This mode of suppression seems to directly interfere with the cellular CI mechanism, which is mediated by the phage WO genes *cifA* (cytoplasmic incompatibility factor A) and *cifB* in *Wolbachia*, the former of which also causes rescue in the infected females [85–89]. For *L. distinguendus*, the genes and processes underlying *sDis*-induced CI have not been uncovered yet, but any involvement of *cif* genes can be excluded due to their absence in *sDis* [27]. This is also true for *Cardinium*-induced CI, the cellular phenotype of which resembles that of *Wolbachia* but also lacks the *cif* genes [90–93].

The spread of the resistance and consequently the reduction or eradication of the CI phenotype in the population [17,19,20,74,75,94] can entail the loss of the endosymbiont [18–20,74,95]. Indeed, we also observed the loss of CAN-*sDis* in one line of CAN during our experiments. On the other hand, the strain BIR, which is currently uninfected in nature and must have lost its infection, is still susceptible to CI. In addition, the frequency of strains carrying *Spiroplasma* seems to be higher in clade B, which does not exhibit CI, than in clade A. Nevertheless, it seems likely that the absence of *sDis* in most strains of clade C could be linked to CI resistance and that infections and resistance cycle within a host clade over evolutionary time, as assumed for CI-inducing *Wolbachia* [19].

### 4.4. Cytoplasmic incompatibility resistance in CAN (clade B) is a male-dependent trait

To examine the involvement of both sexes in CI resistance, CAN females and males were crossed to females and males from STU (clade A). Crosses between uninfected CAN females and infected STU males resulted in CI, demonstrating that uninfected CAN females are unable to counter CI induced by *sDis* in STU males. By contrast, CI was absent in crosses between CAN males infected with STU-*sDis* and uninfected STU females. Thus, unlike STU males [27], infected CAN males do not induce CI in uninfected STU_tet_ females. CI suppression is therefore a male-dependent trait in *L. distinguendus* and does not depend on the females. Thus, effects exerted by or within males are probably the essential factors for the observed variation in the CI phenotypes within *L. distinguendus*.

Our results agree with theoretical models based on the modification/rescue scheme to describe CI. It assumes that a modification in male sperm results in incompatibility with females unless they have a matching rescue system [1]. The models predict mutations reducing or eliminating the endosymbiont-induced modification of sperm in males should be favoured, as males experience fitness costs due to the infection [18,19]. Thereby, infected males would regain compatibility with uninfected females while retaining compatibility with infected females. In addition, for haplodiploid species, models predict selection on male-dependent reduction in CI to be stronger in the male development type of CI, whereby all offspring develop as males [3,66], than the female mortality type [20]. By contrast, mutations conveying CI resistance to females, which would consist in rescue mechanisms employed by the females themselves, are less likely to establish in the population, as only uninfected females would experience their positive effects [19].

So far, only maternal factors influencing the endosymbiont titre in their male offspring [82] and female-related traits [84] have been experimentally identified as host mechanisms that affect presence and strength of CI. To the best of our knowledge, a male-dependent resistance to *Wolbachia*-induced CI, like in our study, was only detected in the haplodiploid mite *Tetranychus urticae*, which also demonstrated female-related effects on resistance [94].

## 5. Conclusion

In summary, our study suggests a single infection event with *Spiroplasma*, termed *sDis*, from the *S. ixodetis* clade in the common ancestor of the *L. distinguendus* species complex. *sDis* seems to cause CI in only one of the currently known *L. distinguendus* species, in clade A. No CI was found in the representatives of clades B and C, although we cannot exclude its presence in other strains of these clades. CAN (clade B) is able to resist CI with a hitherto unknown male-dependent mechanism. In addition to a haplodiploid mite–*Wolbachia* CI system, this is the second overall case of experimental support for theoretical considerations predicting resistance to CI to evolve in males rather than females. We hypothesize that *sDis*, the CI-inducing endosymbiont, infected the common ancestor of the *L. distinguendus* species complex and resistance to CI evolved independently in at least one strain of clade B and possibly in strains of clade C, followed by the loss of *sDis* infections in clade C. This study demonstrates the potential trajectory of CI induction and resistance in *L. distinguendus-sDis* system and may assist to identify the exact mechanism of *sDis*-induced CI and its evolution.

## Data Availability

Raw data from crossing experiments have been submitted to Figshare and are publicly available [96]. Newly generated DNA sequences have been submitted to NCBI and are available under accession numbers PQ887766–PQ887770 and PQ888867–PQ888886. Electronic supplementary material is available online [97].
